# Efficacy and safety of traditional Chinese medicine injections in the treatment of chronic obstructive pulmonary disease: A protocol for systematic review and network meta-analysis

**DOI:** 10.1097/MD.0000000000027324

**Published:** 2021-09-24

**Authors:** Zhongli Sun, Wei Zhao, Kun Yang, Xingying Li, Penglong Yu

**Affiliations:** a Chongqing Three Gorges Medical College, Chongqing, China; b Xindu Hospital of Traditional Chinese Medicine Affiliated to Chengdu Medical College, Sichuan, China.

**Keywords:** chronic obstructive pulmonary disease, COPD, injections, network meta-analysis, protocol, systematic review, traditional Chinese medicine

## Abstract

**Background:**

Chronic obstructive pulmonary disease (COPD) is a widespread, heterogeneous disease characterized by chronic inflammation of the airway and the gradual blockage of air flow due to bronchial obstruction. At present, a large number of traditional Chinese medicine injections (TCMIs) has been applied in the clinical treatment of COPD. However, there is insufficient evidence of evidence-based medicine of the interaction between them. Therefore, the purpose of this study is through the network meta-analysis to evaluate the efficacy and safety of the different TCMIs treatment of COPD, offering reference and evidence for clinical application.

**Methods:**

We will search 7 databases for randomized controlled trials of TCMI for the COPD, including PubMed, the Cochrane Library, EMbase, China National Knowledge Infrastructure, China Biological Medicine, Chinese Scientific Journals Database, and Wan-fang databases, from the date of the establishment of each database to October 31, 2021. The network meta-analysis will be implemented through Aggregate Data Drug Information System 1.16.8 and Stata 13.0 software. Pulmonary function included forced expiratory volume in 1 second (FEV_1_), forced vital capacity (FVC), and FEV_1_/FVC will be the primary outcomes, FEV_1_ as a percentage of the estimated value (FEV_1_%pred), maximal voluntary ventilation (MVV), MVV as a percentage of the estimated value (MVV%pred), 6 minutes walking distance, The St. George's Respiratory Questionnaire score, and safety/adverse event will be evaluated as secondary outcomes. Mean differences or odds ratios will be used for statistical analysis. We will ensure the reliability of the results through node-split model and heterogeneity analysis. In addition, methodological quality will be evaluated based on the Cochrane Collaboration's tool, and the quality of evidence will be evaluated according to the Grading of Recommendations Assessment, Development and Evaluation system.

**Results:**

This study will provide reliable evidence for the clinical selection of TCMI in the treatment of COPD.

**Conclusion:**

The results of this study will evaluate the efficacy and safety of TCMI in the treatment of COPD, and provide decision-making references for future clinical and scientific research.

## 1 Introduction

Chronic obstructive pulmonary disease (COPD) is a widespread, heterogeneous disease characterized by chronic inflammation of the airway and the gradual blockage of air flow due to bronchial obstruction.^[1]^ The pathogenesis of COPD is closely related to protease/anti-protease imbalance, inflammation, oxidative stress, and mucus hypersecretion.^[2]^ In 2005, COPD caused more than 3 million deaths and is considered one of the third leading causes of death, accounting for 6% of all deaths worldwide.^[3,4]^ In addition, globally, as one of the causes of chronic morbidity and death, most patients with COPD for many years die from the disease itself or complications caused by it. Cough and sputum are the most common symptoms of COPD, occurring in about 30% of patients.^[5]^ At present, the treatment measures of COPD are mainly to relieve patients’ symptoms, reduce exacerbations, improve patients’ quality of life, and exercise tolerance.^[6–8]^

Existing evidence method of traditional Chinese medicine in the treatment of COPD has gained rich experience, and combining traditional Chinese and western medicine treatment can further improve the prognosis of patients.^[9,10]^ Traditional Chinese medicine injections (TCMIs) treating COPD at present mainly include Tanreqing injection (TRQI), Danhong injection (DHI), Xuebiqing injection (XBQI), Shenmai injection (SMI), Huangqi injection (HQI), etc.^[11]^ Studies have confirmed that XBQI can regulate the levels of SP-D and CCL18 in patients with COPD and alleviate the clinical symptoms of patients.^[12]^ TRQI can inhibit the release of tumor necrosis factor-α (TNF-α), IL-1β, interleukin-6 (IL-6), and IL-8, and reduce airway inflammation.^[13]^

At present, a large number of TCMIs has been applied in the clinical treatment of COPD. However, there is insufficient evidence of evidence-based medicine of the interaction between them. Therefore, the purpose of this study is through the network meta-analysis (NMA) to evaluate the efficacy and safety of the different TCMIs treatment of COPD, offering reference and evidence for clinical application.

## 2 Methods

### 2.1 Protocol and registration

The NMA protocol has been registered on the Open Science Framework platform (https://osf.io/dkgcq), registration number: DOI 10.17605/OSF.IO/DKGCQ. This protocol follows the Preferred Reporting Items for Systematic Reviews and Meta-Analyses Protocols guidelines.^[14]^

### 2.2 Ethics

Since NMA does not involve the collection of private information, this research does not require ethical approval.

### 2.3 Eligibility criteria

The participant (P), intervention (I), comparator (C), outcome (O), and study design (S) are the 5 main factors determining the inclusion and exclusion criteria of this research.

#### 2.3.1 Type of study design

This study is a systematic review with NMA of randomized controlled trials (RCTs) on TCMI for the COPD. All relevant RCTs using TCMI for the COPD will be included. Quasi-RCTs will be excluded such as those allocating by medical record number. The specific participants, interventions, comparators, outcomes criteria are as follows.

#### 2.3.2 Type of participant

All patients met the diagnostic criteria recommended by the Global Initiative for Chronic Obstructive Pulmonary Disease^[15]^: Patients with COPD whose forced expiratory volume in 1 second (FEV_1_)/forced vital capacity (FVC) after bronchodilator inhalation were less than 70% were defined as COPD, and the condition was stable/acute exacerbation. Patients with other serious complications were not included, and the course and severity of the disease were approximately the same regardless of sex, age, nationality, or educational background.

#### 2.3.3 Type of interventions and comparators

Treatment group TCMI in combination with western medicine or taking TCMI alone, the control group did not intervene, placebo or western medicine. TCMI include TRQI, Reduning injection, XBQI, SMI, Shenfu injection, DHI, HQI, etc.

#### 2.3.4 Type of outcomes

##### 2.3.4.1 Primary outcomes

(1) FEV_1_;
(2) FVC;
(3) FEV_1_/FVC;

##### 2.3.4.2 Secondary outcomes

(1) FEV_1_ as a percentage of the estimated value (FEV_1_%pred), maximal voluntary ventilation (MVV), MVV as a percentage of the estimated value (MVV%pred), 6 minutes walking distance^[16]^;
(2) The St. George's Respiratory Questionnaire score^[17]^;
(3) Serum inflammatory factors include superoxide dismutase, IL-6, and TNF-α;
(4) Safety and adverse event monitoring.

### 2.4 Literature retrieval strategy

Computer retrieval of published RCTs of TCMI for the COPD is conducted in PubMed, the Cochrane Library (issue 10, 2021), EMbase, China National Knowledge Infrastructure, China Biological Medicine, Chinese Scientific Journals Database, and Wan-fang databases. The time limit of document retrieval is from the establishment of each database to October 31, 2021. The language is limited to English and Chinese. In addition, inclusive literature from the field and references from previous evaluations will be manually retrieved to find other potentially relevant articles. Search terms mainly include: “chronic obstructive pulmonary disease”, “chronic obstructive lung disease”, “COPD”, “traditional Chinese medicine”, “injection”, “Tanreqing”, “Danhong”, “Huangqi”, “Shenmai”, “Reduning”, “Xuebijing”, etc. Taking PubMed as an example, the initial retrieval strategy is shown in Table 1 and will be adjusted according to the specific database.

**Table 1:** Search strategy of the PubMed.

Number	Search terms
#1	Chronic Obstructive Pulmonary Diseases[Mesh]
#2	Chronic Obstructive Pulmonary Diseases[Title/Abstract] OR Chronic Obstructive Lung Disease[Title/Abstract] OR COAD[Title/Abstract] OR COPD[Title/Abstract] OR Chronic Obstructive Airway Disease[Title/Abstract] OR Chronic Obstructive Pulmonary Disease[Title/Abstract] OR Airflow Obstruction, Chronic[Title/Abstract]
#3	#1 OR #2
#4	Traditional Chinese medicine[Title/Abstract]
#5	Injections[Title/Abstract]
#6	Tanreqing[Title/Abstract] OR Danhong[Title/Abstract] OR Huangqi[Title/Abstract] OR Shenmai[Title/Abstract] OR Reduning[Title/Abstract] OR Xuebijing[Title/Abstract] OR Shenfu[Title/Abstract]
#7	#5 OR #6
#8	randomized controlled trial[Publication Type]
#9	controlled clinical trial[Publication Type]
#10	randomized[Title/Abstract]
#11	randomly[Title/Abstract]
#12	#8 OR #9 OR #10 OR #11
#13	#3 AND #4 AND #6 AND #12

### 2.5 Literature selection and data extraction

**Figure 1:**
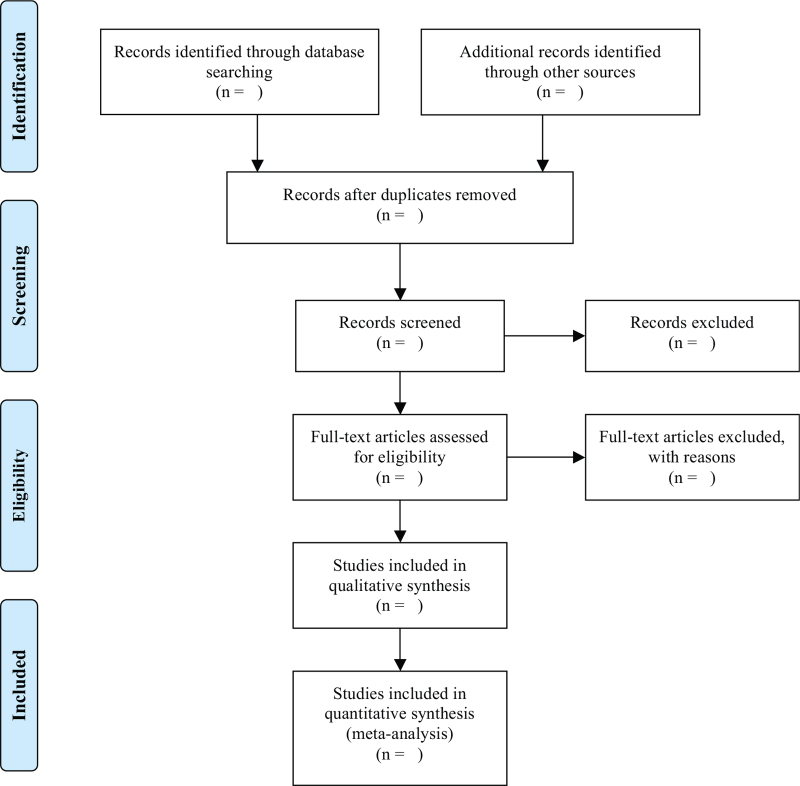
PRISMA flow diagram of the study selection process.

As shown in Figure 1, Zhongli Sun and Wei Zhao will independently screen literatures according to inclusion and exclusion criteria: After importing the retrieved literature into EndNote X9.0 (Stanford, Connecticut, https://endnote.com), the duplicate literature was eliminated; Conduct a preliminary screening by reading the headline summary to exclude literature that does not meet the inclusion criteria; Reading the full text and making final selections; Data extraction using a pre-designed data extraction table for the included literature and cross-checking the results; In case of disagreement, the third researcher Kun Yang will be called upon to assist in judgment. Microsoft Excel (Redmond, Washington, https://www.microsoft.com/zh-cn) will be used to extract the data and collecting relevant information. Data extraction mainly included basic information of the literature (first author name, year of publication), basic information of study subjects (gender, average age, sample size, information of intervention and control group, intervention time, results, and follow-up time). The outcomes included FEV_1_, FVC, FEV_1_/FVC, FEV_1_%pred, MVV, MVV%pred, 6 minutes walking distance, St. George's Respiratory Questionnaire score, superoxide dismutase, IL-6, TNF-α, AR, etc. At the same time, the key factors of bias risk assessment are extracted. We will contact the corresponding authors for additional information if necessary.

### 2.6 Risk of bias assessment

Methodological quality will be assessed based on the bias tool (ROB) in Cochrane Handbook 5.1.0. Two trained researchers Zhongli Sun and Xingying Li will independently evaluate the risk of bias of the included studies. In case of dispute, submit to corresponding author Penglong Yu for arbitration. Cochrane bias risk assessment tool will be used to assess the risk of RCTs being included in NMA, 7 items are included^[18]^: random sequence generation, allocation concealment possibility; blinding of participants and personnel; blinding of outcome assessment; incomplete outcome data; selective reporting; and other bias. Based on the above 7 entries, the judgment is divided into 3 levels: low risk of bias, high risk of bias, and unclear risk of bias.

### 2.7 Data synthesis and statistical methods

#### 2.7.1 Network meta-analysis

This study will use Aggregate Data Drug Information System 1.16.8 for NMA.^[19]^ Based on Bayesian framework, the software uses Markov Chain-Monte Carlo algorithm to prior evaluate and process the extracted data, which provides support for further research and decision making. Preset model parameters: 4 chains will be used for simulation analysis, with an initial value of 2.5, a step size of 10, 20,000 annealing times, and 50,000 simulation iterations. Mean differences or odds ratios will be used as the effect sizes for statistical analysis, both with 95% credible intervals. Firstly, the network evidence plot is generated according to different outcome indicators. The network evidence plot consists of boxes and lines. The box represents each intervention included in the analysis, while the line represents RCT evidence for a direct comparison between the 2 interventions, and the number represents the number of studies for the direct comparison. According to the results of the NMA, rank probability plot of various modeling methods is generated and sorted by dominance, with Rank top1 being the optimal sequence. However, if the lower score is better in the score standard of the outcome, the lowest ranked prediction sequence is the optimal sequence.

#### 2.7.2 Statistical model selection

Node-split model will be used to verify the consistency of the corresponding data. If there is no statistical difference (*P > *.05) between direct comparison and indirect comparison, the consistency model is used, whereas the inconsistency model will be used for analysis. If the consistency model is adopted, then the stability of the results is verified by the inconsistency model: when the inconsistency factors including 0, and at the same time inconsistency standard deviation including 1 says the result of consistency model is more stable and reliable. At the same time, various analysis models are iterated with preset parameters, and the convergence of iteration effect will be judged by potential scale reduced factor (PSRF). When the PSRF value is close to or equal to 1 (1 ≤ PSRF ≤ 1.05), it indicates that the convergence is complete, the model has good stability, and the conclusion of analysis is reliable. If the PSRF value is not in this range, the iteration continues manually until the PSRF value reaches the range standard.

#### 2.7.3 Heterogeneity test

Before the combination of effect size, the heterogeneity of the included literature is tested using STATA 15.0 software (Stata Corp, College Station, TX, https://www.stata.com). Evaluate the heterogeneity between studies through I^2^. When I^2^ > 50%, it indicates that the heterogeneity between studies is large, using a random effect model; when I^2^ < 50%, it indicates that the heterogeneity between studies is small or there is no difference qualitative, using a fixed effect model. When the heterogeneity is greater, the source of heterogeneity should be further sought.

#### 2.7.4 Sensitivity analysis

If necessary, sensitivity analysis will be used to assess the impact of the studies on the random effects model. After each study was excluded one by one, the data analysis was carried out again to determine the stability of the results. If there is no qualitative change in the combined effect showed in the results, the results are stable.

#### 2.7.5 Subgroup analysis

If there is clinical and methodological heterogeneity, we will conduct a subgroup analysis of the patient's age, the degree and stage of COPD, smoking history, duration of treatment, or study quality.

#### 2.7.6 Small sample effect/publication bias

If 10 or more studies are included in the NMA, a comparison-adjusted funnel plot is developed using Stata to evaluate the presence of small sample effects or publication bias in the intervention network. If the plot is asymmetric and there is no inverted funnel shape, it indicates that there may be publication bias. The reasons may be related to the small sample size, allocation concealment, and insufficient implementation of blind method.

#### 2.7.7 Dealing with missing data

If the literature information is clearly incorrect or incomplete, we will contact the first author or the first author of the literature via email address. If no response is received, the document should be deleted.

#### 2.7.8 Evaluating the quality of the evidence

To grade evidence quality and understand the current situation of evidence rating thereby analyzing possible problems, The Grading of Recommendations Assessment, Development and Evaluation instrument will be used to assess the quality of evidence in the NMA.^[20]^ Based on bias, inconsistent, inaccurate, indirect, and the risk of publication bias 5 degradation factors, the quality classification for the 4 level of evidence: high, medium, low, and very low.

#### 2.7.9 Patient and public involvement

There was no patient or public involvement in the preparation of this protocol.

## 3 Discussions

In recent years, more and more RCTs of TCMIs in the treatment of COPD, and studies have confirmed that TCMIs play an important role in the application of effective treatment of COPD. However, these results have not been fully confirmed. Therefore, this NMA was designed to evaluate the efficacy and safety of TCMI in the treatment of COPD. The purpose of this study is to provide a basis for clinical practice of traditional Chinese medicine, as well as to help doctors make clinical decisions for the treatment of COPD patients and strengthen the treatment strategy of COPD. Our study used a comprehensive analysis of all RCTs of TCMIs for COPD, including SMI, XBQI, DHI, HQI, etc, to synthesize the evidence and rank the available TIMIs for COPD. There are some potential limitations to this study, such as the fact that most studies published in Chinese journals do not have a strictly randomized design. In addition, due to the limited ability of the authors, only English and Chinese literature was retrieved, which may create a risk of bias.

## Author contributions

**Conceptualization:** Zhongli Sun, Wei Zhao, Penglong Yu.

**Data curation:** Zhongli Sun, Wei Zhao, Kun Yang.

**Formal analysis:** Zhongli Sun, Wei Zhao.

**Funding acquisition:** Kun Yang.

**Methodology:** Zhongli Sun, Wei Zhao, Xingying Li.

**Project administration:** Zhongli Sun, Wei Zhao, Kun Yang.

**Writing – original draft:** Zhongli Sun, Wei Zhao.

**Writing – review & editing:** Zhongli Sun, Wei Zhao, Kun Yang, Xingying Li, Penglong Yu.
